# Electrospun Scaffolds as Cell Culture Substrates for the Cultivation of an In Vitro Blood–Brain Barrier Model Using Human Induced Pluripotent Stem Cells

**DOI:** 10.3390/pharmaceutics14061308

**Published:** 2022-06-20

**Authors:** Felix Rohde, Karin Danz, Nathalie Jung, Sylvia Wagner, Maike Windbergs

**Affiliations:** 1 Institute of Pharmaceutical Technology and Buchmann Institute for Molecular Life Sciences, Goethe University Frankfurt, Max-von-Laue-Str. 9, 60438 Frankfurt am Main, Germany; f.rohde@em.uni-frankfurt.de (F.R.); n.jung@em.uni-frankfurt.de (N.J.); 2 Department Bioprocessing & Bioanalytics, Fraunhofer-Institute for Biomedical Engineering IBMT, Joseph-von-Fraunhofer-Weg 1, 66280 Sulzbach, Germany; karin.danz@ibmt.fraunhofer.de (K.D.); sylvia.wagner@ibmt.fraunhofer.de (S.W.)

**Keywords:** electrospinning, blood–brain barrier, human induced pluripotent stem cells, tissue engineering, extracellular matrix, basement membrane, in vitro model, crosslinking, gelatin, polycaprolactone

## Abstract

The human blood–brain barrier (BBB) represents the interface of microvasculature and the central nervous system, regulating the transport of nutrients and protecting the brain from external threats. To gain a deeper understanding of (patho)physiological processes affecting the BBB, sophisticated models mimicking the in vivo situation are required. Currently, most in vitro models are cultivated on stiff, semipermeable, and non-biodegradable Transwell^®^ membrane inserts, not adequately mimicking the complexity of the extracellular environment of the native human BBB. To overcome these disadvantages, we developed three-dimensional electrospun scaffolds resembling the natural structure of the human extracellular matrix. The polymer fibers of the scaffold imitate collagen fibrils of the human basement membrane, exhibiting excellent wettability and biomechanical properties, thus facilitating cell adhesion, proliferation, and migration. Cultivation of human induced pluripotent stem cells (hiPSCs) on these scaffolds enabled the development of a physiological BBB phenotype monitored via the formation of tight junctions and validated by the paracellular permeability of sodium fluorescein, further accentuating the non-linearity of TEER and barrier permeability. The novel in vitro model of the BBB forms a tight endothelial barrier, offering a platform to study barrier functions in a (patho)physiologically relevant context.

## 1. Introduction

Neurovascular and neurological diseases constitute an enormous social and economic burden worldwide and are expected to increase further in the coming decades in light of the aging population [1,2]. Accordingly, efforts to understand and treat neurological diseases are undertaken in basic research and through translational approaches in the pharmaceutical industry. However, the efficient transport of potential therapeutics from the bloodstream to the brain is hampered by the physiological characteristics of the blood–brain barrier (BBB), which constitutes a tightly closed cellular interface between microvasculature and the central nervous system in the human body. Its specialized architecture comprises endothelial cells, pericytes, and astrocytes, which adhere to a basement membrane, a thin fibrillary extracellular matrix (ECM) layer [3]. This basement membrane mainly consists of collagen-type IV fibrils, laminin, heparan sulfate proteoglycans, and nidogens, providing physical support to the adhering cells and regulating the cellular crosstalk between the endothelial layer and the surrounding neural cells [4,5,6]. By forming the tightest endothelial barrier in the body, the BBB is a dynamic, highly effective, and selective barrier, limiting the exchange of molecules and thereby maintaining homeostasis of the central nervous system and preventing the entry of pathogens and neurotoxic substances [7].

Overcoming this barrier for the therapeutic application of active pharmaceutical ingredients to the brain constitutes a major focus of pharmaceutical research. Despite interspecies differences in the expression of relevant receptors and transporters, studies have mainly been carried out using animal models [8]. To overcome these limitations, research efforts in the development of in vitro BBB models have notably been increased to enable the investigation of neurovascular diseases and drug transport across this endothelial barrier in humanized systems. The experimental setup for these in vitro models usually comprises the cultivation of endothelial cells in Transwell^®^ membrane inserts, representing an extremely simplified version of the in vivo BBB microenvironment [9,10]. So far, immortalized cell lines have been used, combining easy handling with high reproducibility of experimental conditions and obtained results. However, cell lines are prone to experience dedifferentiation associated with the loss of essential cellular functions (e.g., reduction in tight junctions and subsequent decrease in barrier integrity). When modeling the human BBB using cell lines, transendothelial electrical resistance (TEER) values typically lie below 200 Ω×cm^2^, not adequately mimicking the barrier properties in vivo, for which TEER values of more than 2000 Ω×cm^2^ are reported [8,11,12]. In order to cultivate in vitro models with higher physiological relevance, primary cells can be isolated from animals, which exhibit improved phenotype retention, resulting in BBB models with low permeability and good overall functionality [11,13]. However, the isolated primary cells show considerable variability and further exhibit interspecies discrepancies regarding physiology on a cellular level, limiting their use in predictive in vitro models [14,15]. Overcoming these drawbacks, human induced pluripotent stem cells (hiPSCs) have emerged as a promising source of reproducible human-derived biological material, showing great potential for the development of robust BBB in vitro models [15,16,17]. In comparison to primary cells, hiPSCs are able to proliferate indifferently and further differentiate into specialized human brain capillary endothelial cells, which are requisite for forming a functional BBB. Although hiPSCs represent a powerful tool for mimicking human physiology, their potential is often limited by cultivation in a very artificial environment using Transwell^®^ cell culture inserts with stiff and flat membranes. The importance of the native extracellular matrix (ECM) in tissue development is often neglected, although this non-cellular component provides physical support to cells and tissues and plays an integral role in providing biochemical and biomechanical cues for cell differentiation. In contrast to common artificial growth substrate, the ECM is a highly dynamic structure that is constantly being remodeled by the resident cells [18]. Differing from the conditions provided by the native ECM, commercial cell culture inserts comprise a membrane made of synthetic polymers, such as polycarbonate, polytetrafluoroethylene, or polyethylene terephthalate, preventing dynamic interaction with cells. Therefore, cells that have been cultivated on top of these stiff membranes cannot restructure their surroundings and tend to form abnormal cell–cell interactions compared to in vivo tissues, leading to artificial intracellular functions and polarity [17,18]. Furthermore, compared to the basement membrane, these inserts are thicker, less porous, mechanically stiffer, lack surface roughness, and the nanoporous, three-dimensional structure of collagen fibrils found in the native tissue [19]. It has been shown that cells respond differently on softer substrates, representing the human ECM more closely compared to harder substrates such as Transwell^®^ membrane inserts or glass [20,21,22,23]. These studies highlight the complex interplay between substrate stiffness and induced cell response, which is crucial for simulating the in vivo state. Therefore, to cultivate physiologically relevant in vitro models of the BBB, it is important to provide hiPSCs with an adequate culture substrate that can be dynamically degraded and remodeled to promote physiological differentiation.

To address this challenge, we developed a nanofibrous substrate for the cultivation of an hiPSC-derived in vitro model of the BBB mimicking the adaptive nature of the human basement membrane. The rational design of the scaffolds entailed the tuning of biophysical properties, including surface wettability, fiber diameter, and porosity, which were tailored to meet the requirements of hiPSCs cultivation. The barrier integrity of the formed BBB was assessed via visualization of tight junction proteins, measurement of the barrier integrity as a function of TEER values, and paracellular permeability of sodium fluorescein.

## 2. Materials and Methods

### 2.1. Materials and Chemicals

Polyvinylpyrrolidone (PVP) (1,300,000 g/mol), poly-ε-caprolactone (PCL) (80,000 g/mol), gelatin type A from porcine skin (bloom 300 g), platelet-poor plasma-derived human serum, retinoic acid, and aqueous glutaraldehyde solution (GA) (25% *v*/*v*) were obtained from Sigma Aldrich (Darmstadt, Germany). Glacial acetic acid, sodium fluorescein, and polycarbonate Transwell^®^ inserts with 3.0 µm pores were purchased from VWR Chemicals (Darmstadt, Germany). Single-use syringes and needle tips were acquired from B. Braun (Melsungen, Germany). Trifluoroethanol (TFE, purity > 99.8%) was obtained from Thermo Fisher Scientific (Dreieich, Germany). Glycine was sourced from Merck Millipore (Darmstadt, Germany). Dulbecco’s phosphate-buffered saline (PBS) was purchased from Biowest (Nuaille, France). RPMI 1640, fetal calf serum (FCS), penicillin, streptomycin, GlutaMAX, sodium pyruvate, non-essential amino acids, MEM vitamin solution, mercaptoethanol, human endothelial serum-free growth medium (heSFM), alamarBlue^®^, propidium iodide, fluorescein diacetate, sodium fluorescein, paraformaldehyde (PFA), Triton-X-100, Accutase^®^, Immu-Mount™ medium, bovine serum albumin (BSA), DMEM/F12 medium, and NucBlue™ Fixed Cell ReadyProbes™ were obtained from Fisher Scientific (Schwerte, Germany). Human brain microvascular endothelial cells were kindly provided by Kwang Sik Kim (John Hopkins University, Baltimore, MD, USA), and the human induced pluripotent stem cell line Ukki011-A was provided by the European Bank for Induced pluripotent Stem Cells (EBiSC, IMI project, www.ebisc.org, accessed on 30 May 2022), which is also registered on hpsreg.eu and under BioSample ID SAMEA2774610. The EBiSC lines are fully consented for research use and quality controlled. At the point of banking with EBiSC, sterility (absence or mycoplasma, microbial growth), cell line identity based on short tandem repeat assay, as well as karyotyping are routinely performed; these data and accompanying Certificates of Analysis are available on request. Additionally, mycoplasma testing was performed on a regular basis. Stem cell medium mTeSR1 was obtained from STEMCELL Technologies (Vancouver, BC, Canada), and basic fibroblast growth factor (bFGF) was purchased from R&D Systems (Minneapolis, MN, USA). Matrigel^®^ was acquired from Corning (Amsterdam, The Netherlands). CellCrown™ inserts were purchased from Scaffdex Oy (Tampere, Finland). Anti-occludin was purchased from Santa Cruz Technologies (sc-133256, Santa Cruz, CA, USA), and goat anti-mouse IgG Alexa Fluor^®^ 555 was obtained from Fisher Scientific (A28180, Schwerte, Germany). All listed chemicals were of at least analytical grade and used without further purification.

### 2.2. Preparation of Solutions and Electrospinning

All cell culture scaffolds were electrospun on a water-soluble base layer, which was created based on a PVP (10% *w*/*v*) solution in ethanol. Gelatin (12% *w*/*v* in 90% *v*/*v* acetic acid) and PCL (13% *w*/*v* in TFE) solutions were used to fabricate the cell substrates. All solutions were prepared under stirring conditions at room temperature. Before electrospinning, all solutions were transferred into a 5 mL single-use syringe equipped with a 27 G blunted needle tip. The formulations were electrospun in a custom-built spinning cabinet with a UV-disinfected chamber with environmental controls set to 20% relative humidity and 30 °C. For each cell culture scaffold, a PVP base layer was electrospun with a flow rate of 5.0 mL/h at 10 kV. The scaffolds were subsequently spun onto the PVP layer by pumping either gelatin solution with a flow rate of 0.6 mL/h at 15.4 ± 2.2 kV (herein referred to as gelatin) or PCL solution with a flow rate of 0.6 mL/h at 9 ± 1.5 kV (herein referred to as PCL). Additionally, a hybrid fiber mat was created by parallel electrospinning gelatin and PCL solutions with the above-mentioned parameters (hereafter referred to as hybrid). For visualization of PCL fibers by fluorescence microscopy, 2 mg of sodium fluorescein was added to a PCL (13% *w*/*v* in TFE) solution and electrospun with a flow rate of 0.6 mL/h at 14 ± 4 kV. All solutions were electrospun onto a rotating drum cylinder with a diameter of 10 cm at 50 revolutions per minute and stored in a desiccator until further use.

### 2.3. Crosslinking of Electrospun Fibers

Crosslinking of gelatin fibers was carried out by exposing the fiber mats to the saturated vapor phase of glutaraldehyde (5%, 15%, and 25% (*v*/*v*)) at room temperature for 60, 90, and 120 min, respectively.

### 2.4. Scanning Electron Microscopy and Analysis of Fiber Mats

Scanning electron microscopic images of all nonwoven fiber mats were obtained on a field emission scanning electron microscope (S 4500, Hitachi, Chiyoda, Japan) at 5 kV. Before image acquisition, samples were gold-sputtered (Agar Sputter Coater, Agar Scientific, Stansted, UK). Fiber diameter analysis was conducted on 100 randomly chosen fibers using Fiji [24]. Pore size was evaluated using Fiji and by thresholding of the obtained SEM images, excluding the fibers and measuring the area of pores.

### 2.5. Confocal Raman Microscopy

Chemically selective visualization of fiber mats was conducted via confocal Raman microscopy using an alpha 300R+ from WITec (Ulm, Germany) coupled with a 532 nm laser. Using a laser power of 40 mW, a 50x objective (NA 0.8, Epiplan Neofluar, Zeiss, Germany), and a confocal pinhole to reject out-of-focus signals, Raman image scans were acquired with an integration time of 0.2 s and a resolution of 0.5 µm. Raman spectral data sets were treated with a cosmic ray removal procedure, and background subtraction was performed using the shape function of FOUR software (WITec, Ulm, Germany). Spectra were further processed via a supervised k-means cluster and subsequent basis analysis to generate false-color image scans.

### 2.6. Attenuated Total Reflection-Infrared (ATR-IR) Spectroscopy

Infrared spectra were obtained using an ALPHA II Platinum FT-IR spectrometer (Bruker, Billerica, MA, USA). For every data point, 24 spectra were recorded in a shift range of 3100–400 cm^−1^ at a resolution of 4 cm^−1^ and averaged and normalized using OPUS software version 8.1.29 (Bruker, Billerica, MA, USA).

### 2.7. Thickness of Fiber Scaffolds

The thickness of the fiber mats was assessed using a coating thickness gauge equipped with an F5 sensor (MiniTest 735, ElektroPhysik Dr. Steingroever GmbH & Co. KG, Cologne, Germany). Fiber mats were placed on stainless steel and covered with a tared microscopy coverslip, and five measurements were carried out to determine the thickness of the fiber mat. Cell substrate thickness was evaluated after electrospinning three different volumes of polymer solutions (0.5 mL, 1.0 mL, and 1.5 mL).

### 2.8. Tensile Testing

The mechanical properties were analyzed using a single-column tensile tester (5942, Instron, Norwood, OH, USA) with a 50 N load cell. Electrospun scaffolds were cut into rectangular shapes (1.0 cm × 4.6 cm (width × length)). Next, 50 mN pre-stress was applied to each sample to ensure testing in a reproducible manner. To assess the mechanical properties of the electrospun fiber mats in cell culture media, all scaffolds were soaked for 30 min in PBS at a temperature of 37 °C, ensuring a complete wetting, and were subsequently exposed to 5 mN of pre-stress before measurement. Mechanical testing of the scaffolds was carried out with a traveling speed of 10 mm/min. The distance and force were recorded simultaneously. Bluehill Universal software (Instron, Norwood, MA, USA) was used to calculate tensile stress and strain in dependence of the fiber mat thickness. Young’s modulus was determined by calculating the slope of the linear region. All experiments were carried out in triplicate.

### 2.9. Surface Wettability

Dynamic contact angle measurements were carried out on an optical contact angle measurement device (OCA 15LJ, Dataphysics, Filderstadt, Germany). Samples were clamped into a designated paper mount, and a needle tip (diameter of 0.31 mm) was used to dispense a volume of 2 µL double distilled water. The droplets were applied to the fiber mat surface, and absorption was recorded with a frame rate of 10 Hz (PCL) and 90 Hz (gelatin and hybrid fiber mats). Experiments were conducted in triplicate.

### 2.10. Quantification of Released Sodium Fluorescein from Electrospun Fibers

For the quantification of fluorescein release from electrospun fibers, a 10 mm circular fiber mat punch was incubated in 3 mL PBS for 7 days at 37 °C under shaking conditions. A calibration curve of fluorescein was prepared, covering a range from 200 to 1000 ng/mL in PBS. All measurements were carried out in threefold determination in a plate reader (Spark., Tecan Germany GmbH, Crailsheim, Germany) at an excitation wavelength of 485 nm and an emission wavelength of 535 nm.

### 2.11. Preparation of Electrospun Fibers for Cell Cultivation

To avoid adverse cell reactions, all scaffolds were incubated for 4 h with a 2 M (15% *w*/*v*) glycine solution to neutralize residual GA from the crosslinking procedure. Subsequently, the electrospun scaffolds were washed twice with PBS and soaked in cell culture media.

### 2.12. Cultivation of Immortalized Human Brain Microvascular Endothelial Cells

Immortalized human brain microvascular endothelial cells (hBMEC) were cultivated in RPMI 1640 supplemented with 20% FCS, 100 U/mL penicillin, 100 µg/mL streptomycin, 2 mM GlutaMAX, 1 mM sodium pyruvate, 1% non-essential amino acids, and 1% MEM vitamin solution. The hBMECs were seeded with a density of 1 × 10^5^ cells/cm^2^ on the electrospun fiber scaffolds mounted in CellCrown™ inserts. Commercially available polycarbonate Transwell^®^ inserts with a 3 µm pore diameter were used as control substrates. Experiments were conducted 48 h after seeding.

### 2.13. Cultivation and Differentiation of Human Induced Pluripotent Stem Cells

The differentiation of human induced pluripotent stem (hiPS) cell-derived brain capillary endothelial-like cells (BCECs) was carried out according to Danz et al. [17]. The hiPS cell line Ukki011-A was seeded with a density of 5 × 10^3^ cells/cm^2^ on Matrigel^®^-coated 6-well plates and cultivated for 3 days in mTeSR1 medium before differentiation. On day 0, mTeSR1 medium was changed to unconditioned medium (DMEM/F12 with 20% knock-out serum replacement, 1% non-essential amino acids, 0.5% GlutaMAX, and 0.1 µM 2-mercaptoethanol) and exchanged for 5 consecutive days. On the 6th day, the cell culture medium was changed to endothelial medium supplemented with growth factors (heSFM with 1% platelet-poor plasma-derived human serum (PDS), 20 ng/mL bFGF, and 10 µM retinoic acid). On day 8, hiPS-BCECs were dissociated with Accutase^®^ for 25 min at 37 °C and seeded with a density of 1 × 10^6^ cells/cm^2^ onto the fiber mats mounted in CellCrown™ inserts or onto Transwell^®^ inserts with a pore size of 3 µm coated with a mixture of 400 µg/mL human collagen IV and 100 µg/mL fibronectin in 0.05% acetic acid. After 24 h, the medium was changed to endothelial medium without growth factors (heSFM with 1% PDS) in the basolateral compartment and to enriched endothelial medium (heSFM with 5% PDS) on the apical side. The cells were cultivated for at least 48 h until they reached the desired TEER values before conducting further experiments.

### 2.14. Evaluation of Cell Viability

The cell viability of hBMECs and hiPS-BCECs was determined by conducting live–dead staining and using an alamarBlue^®^ assay. Live–dead stainings were performed by replacing the cell culture medium with a solution containing 40 µg/mL propidium iodide and 25 µg/mL fluorescein diacetate in PBS for 1 min. Afterward, the cells were washed with PBS twice and immediately imaged using an Olympus IX 70 microscope (Olympus Corporation, Tokyo, Japan). AlamarBlue^®^ assays were carried out by replacing the cell culture medium with 10% (*v*/*v*) alamarBlue^®^ reagent diluted in the cell culture medium and subsequent incubation for 3 h at 37 °C. Afterward, the cell culture medium was transferred to clear-bottom 96-well plates, and the fluorescence intensity was measured using a plate reader (Tecan Infinite 200, Tecan Group Ltd., Männedorf, Switzerland) at an excitation wavelength of 560 nm and an emission wavelength of 610 nm. The background fluorescence of the cell culture medium was subtracted using Microsoft Office Excel 2016 (Redmond, WA, USA). Cells cultivated on commercial inserts were used as the positive control. All measurements were carried out in triplicate.

### 2.15. Immunofluorescence Staining

The hiPS-BCECs grown on the electrospun fiber scaffolds were fixed with a 10% (*v*/*v*) paraformaldehyde solution for 10 min. Afterward, the cell monolayer was washed with PBS, protein binding sites were blocked, and cells were permeabilized with 2% (*w*/*v*) BSA and 0.2% (*v*/*v*) Triton X-100 in PBS for 1 h at ambient temperature. The primary antibody anti-occludin was diluted at 1:500 in 0.1% BSA in PBS and incubated overnight at 4 °C. Subsequently, the monolayer was washed with PBS, and the secondary antibody (goat anti-mouse IgG-Alexa Fluor^®^ 555) was diluted to 1:1000 in PBS, and 1 drop of NucBlue™ Fixed Cell ReadyProbes™ reagent was added. The secondary antibody was incubated for 1 h at ambient temperature. The electrospun fibers were removed from the CellCrown™ inserts and mounted with Immu-Mount™ medium, dried overnight, and imaged using a confocal microscope (TCS SP8, Leica Microsystems, Wetzlar, Germany).

### 2.16. Impedance Spectroscopy

Impedance spectroscopy (cellZscope, nanoAnalytics, Münster, Germany) was used to determine the transendothelial electrical resistance (TEER) of the formed cellular monolayer. To this end, hiPS-BCECs were cultivated in the cellZscope module at 37 °C, 5% CO_2_, and 90% relative humidity. TEER measurements were carried out every 12 h over the course of the 72 h cultivation period. The obtained data were analyzed using Microsoft Office Excel 2016 and correlated to a Transwell^®^ membrane insert without cells (negative control).

### 2.17. Determination of Permeability Coefficients

The permeability coefficient was determined by adding 1 µg/mL of sodium fluorescein to the apical side of the cultured cellular monolayer and incubated for 4 h at 37 °C. Media were collected from the apical and basolateral side and transferred to a flat-bottom, black 96-well plate optimized for fluorescence measurements (Fisher Scientific, Schwerte, Germany), and the fluorescence intensity was determined using a plate reader (Tecan Infinite 200, Tecan Group Ltd., Männedorf, Switzerland) at an excitation wavelength of 485 nm and emission wavelength of 535 nm. All measurements were conducted in triplicate.

### 2.18. Statistical Analysis

All results are presented as their mean ± standard deviation (SD). Statistically significant differences between experimental groups were analyzed using the Mann–Whitney-U test, which requires no normality of the data and is specifically applied to small data sets. *p*-Values < 0.05 were considered to be statistically significant (*, *p* < 0.01 **). All calculations were performed using Microsoft Office Excel 2016 and Origin Pro 2021.

## 3. Results and Discussion

### 3.1. Fiber Fabrication and Crosslinking

In search of more physiologically relevant in vitro BBB models and to overcome the previously mentioned drawbacks of Transwell^®^ membrane inserts, we designed electrospun polymer scaffolds, creating a three-dimensional fibrous structure to mimic the microenvironment of the basement membrane of the BBB. To this end, polymers were selected to ensure a close resemblance with native collagen fibrils, taking into account fiber diameter, mechanical behavior, and surface wettability. We chose PCL, an FDA-approved, biocompatible, and biodegradable polymer that is insoluble in water, to provide structural stability and flexibility to the electrospun cell scaffolds. To enhance cell adhesion to the fibrous scaffold, we selected gelatin, a degradation product of collagen, to increase the surface wettability of the scaffolds and introduce cell recognition patterns. On the basis of these polymers, we designed three scaffolds, one consisting of pure PCL, one consisting of gelatin, and a third hybrid scaffold combining both polymers with a mass ratio of 1:1.

**Figure 1:**
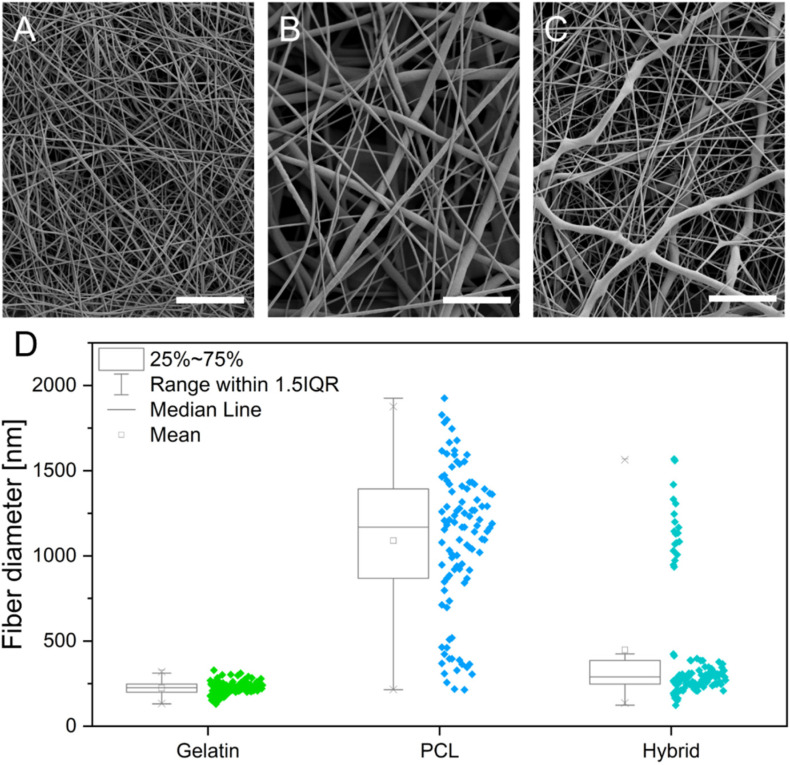
(**A**–**C**) Scanning electron micrographs of electrospun gelatin (**A**), PCL (**B**), and hybrid (**C**) scaffolds. (**D**) Box whisker plots showing the fiber diameter distribution in gelatin, PCL, and hybrid scaffolds. Scale bars represent 10 µm.

The electrospinning process was stable for all formulations and yielded homogeneous fiber mats with randomly orientated, defect-free fibers, displaying a smooth outer surface for gelatin, PCL, and hybrid scaffolds, as revealed by scanning electron micrographs (Figure 1A–C, respectively). Measurements of single electrospun fibers within the scaffolds revealed a diameter distribution of 224 ± 38 nm and 1090 ± 422 nm for gelatin and PCL scaffolds, respectively (Figure 1D). The resulting fiber diameter of the hybrid scaffold shows two distinct diameter populations, which can be assigned to the respective polymers (gelatin 277.21 nm ± 61.0 nm and PCL 1077.0 ± 205.92 nm). The diameter of individual fibers within the scaffold plays an essential role in mitigating the adhesion, migration, and differentiation of stem cells [25]. The observed fiber diameter of the gelatin fibers within the scaffolds is in accordance with the reported fibril diameter of collagen (50–500 nm), constituting the main component in the basement layer of the BBB [26,27]. Consequently, the gelatin nanofibers in the scaffolds promote interaction with cells. A reduction in the fiber diameter further allows for tuning of the pore size of the respective cultivation substrate to obstruct or encourage the migration of cells into the fiber network. With respect to the BBB, small pore sizes and subsequent migration of hiPSCs on the surface of the scaffold are desirable to achieve a tight endothelial barrier [28,29,30]. The electrospun gelatin scaffold has an average pore area of 0.21 µm^2^, whereas the pore area of PCL increased more than 10-fold compared to gelatin to an average pore area of 3.59 µm^2^. The incorporation of gelatin nanofibers in the hybrid scaffold decreased the pore area to 0.88 µm^2^. The porosity and pore size of the electrospun scaffolds increased with increasing fiber diameters. This correlation of fiber diameter and pore size results from the higher packing density of nanofibers compared to microfibers and is in accordance with published literature [31,32]. For the cultivation of a tight endothelial barrier, the use of gelatin as a fiber substrate decreases the pore area, which is desired to obstruct the migration of cells into the scaffold, thereby promoting rapid cell spreading over the surface of the culture substrate. Therefore, the incorporation of gelatin serves multiple purposes by introducing cell recognition patterns, mimicking the size of native collagen fibrils and promoting a guided cell migration, leading to a tighter barrier formation.

**Figure 2:**
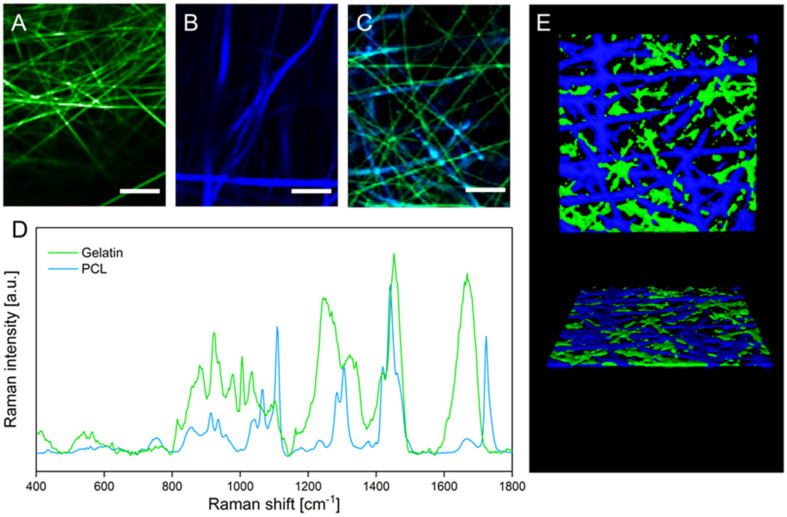
(**A**–**C**) False-color Raman images of gelatin (**A**), PCL (**B**), and hybrid (**C**) scaffolds. (**D**) Raman spectra of gelatin and PCL fibers. (**E**) 3D rendering of confocal Z-stack Raman images visualizing the three-dimensional organization of gelatin and PCL fibers within the hybrid scaffold. Scale bars represent 10 µm.

Although two distinct fiber types in the hybrid scaffold can be distinguished in the scanning electron micrographs, the chemical identity of the respective polymers cannot be confirmed based on these data. To investigate the distribution of the two polymeric fiber types within the hybrid scaffold, the sample was analyzed with confocal Raman microscopy. This laser-based technique enables discrimination of materials based on their chemical composition and therefore the spatial distribution of gelatin and PCL fibers. False-color Raman scans of gelatin and PCL scaffolds show the distribution of the respective fiber types in green and blue (Figure 2A,B). During the analysis of the hybrid scaffold (Figure 2C), both fiber types could be distinguished based on the respective characteristic Raman spectra of gelatin and PCL (Figure 2D). The acquired false-color images display the discrete presence of gelatin and PCL fibers in the hybrid scaffold, approximating the 1:1 mass ratio that was used in the spinning process of the hybrid scaffold. The three-dimensional organization of the two interpenetrating fiber types within the fiber mat is displayed in a 3D rendering in Figure 2E, confirming homogeneous deposition during the electrospinning process of both fiber types, which is necessary to obtain uniform cell migration on the hybrid scaffold.

Because gelatin, as a polypeptide, rapidly dissolves upon contact with aqueous media, further processing of the scaffolds is required to allow for their implementation as cell culture substrates. In order to modify gelatin fibers to be water-insoluble while maintaining their morphology and still providing cell recognition patterns, it is necessary to crosslink the polymer. To evaluate the stabilizing effect of the GA treatment on gelatin nanofibers in aqueous media, we performed a systematic evaluation, varying the percentage of GA and the total duration of crosslinking of scaffolds in the vapor phase (Table 1).

**Figure 3:**
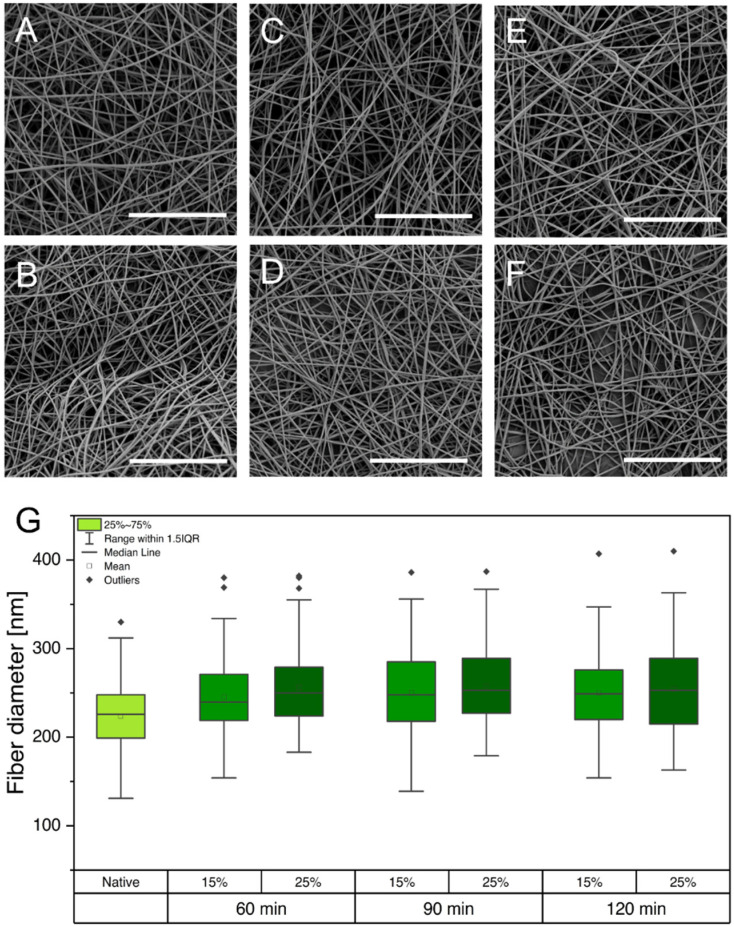
(**A**–**F**) Scanning electron micrographs showing gelatin fibers under various crosslinking conditions: 60 min, 15% (**A**); 60 min, 25% (**B**); 90 min, 15% (**C**); 90 min, 25% (**D**); 120 min, 15% (**E**); and 120 min, 25% (**F**). (**G**) Comparison of diameters of gelatin fibers that were exposed to different crosslinking conditions. Scale bars represent 20 µm.

Crosslinking with 5% (*v*/*v*) GA for all tested time intervals (60, 90, and 120 min) was not sufficient to insolubilize the gelatin nanofibers, leading to immediate dissolution in PBS. However, crosslinking with 15% (*v*/*v*) GA led to semistable gelatin nanofibers that did not dissolve for a maximum of one day. Because the cultivation period of hiPSCs requires the scaffolds to be stable for at least 72 h, the GA percentage was further increased to 25%. Insoluble gelatin nanofibers were only obtained by crosslinking the scaffolds for a minimum of 90 min in the vapor phase of a 25% (*v*/*v*) aqueous GA solution. After evaluating the stability of the gelatin nanofibers in aqueous media, gelatin-based scaffolds were evaluated again using SEM. The preservation of fiber morphology is essential to maintain the porosity of the electrospun scaffolds, enabling sufficient nutrient transport and crosstalk among cells through the scaffold [33]. However, previous studies revealed that crosslinking with GA can promote altered fiber morphology, increased fiber diameter, and film formation, which might obstruct nutrient transport and crosstalk of cells [33,34,35]. Native gelatin fibers (Figure 1A) are compared to samples that were crosslinked under the above-described conditions in scanning electron micrographs shown in Figure 3A–F.

Regardless of the applied GA percentage and crosslinking time, gelatin fibers were able to maintain their nanofibrous morphology. Furthermore, fiber diameters were determined, and no increase was observed for the various crosslinking procedures (Figure 3G). Despite the high atmospheric humidity during crosslinking, the preservation of fiber morphology and diameter is enabled through water absorption by the hydrophilic base layer consisting of PVP (see Supporting Information Figure S1). As a result, the PVP fibers partially dissolve in atmospheric humidity, forming a polymer film that inhibits the wetting and partial dissolution of gelatin fibers, which would cause morphological changes.

**Figure 4:**
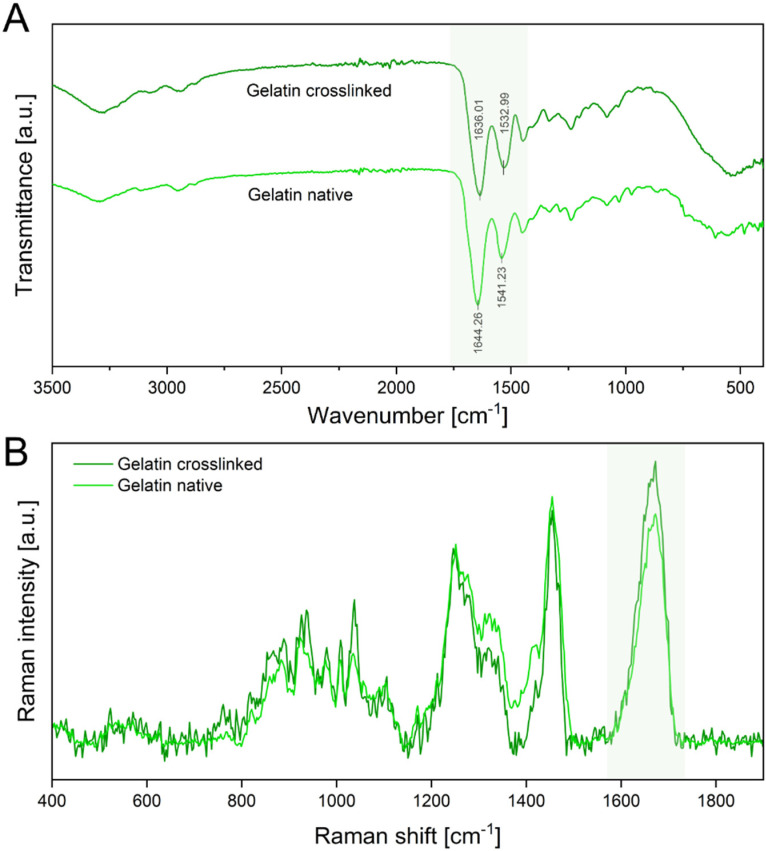
(**A**) ATR-IR spectra of the crosslinked electrospun gelatin fibers and native gelatin fibers. The amide I and amide II peaks are highlighted in green. (**B**) Raman spectra of native and crosslinked gelatin show a distinct C=N stretching vibration at 1664 cm^−1^ of the amide I region, also highlighted green.

Furthermore, GA crosslinks the peptide structure of gelatin by reaction of two aldehyde groups of GA with the amine groups of hydroxylysine or lysine of gelatin, forming Schiff bases [36,37]. The formation of Schiff bases as an indicator for successful crosslinking was confirmed by ATR-IR and Raman spectroscopy (Figure 4A).

ATR-IR spectra show characteristic peaks of amide I (C=O stretching) at 1644 cm^−1^ and amide II (C-H stretch and N-H band) at 1542 cm^−1^ in native gelatin scaffolds. The spectra of crosslinked gelatin show a red shift of the amide I peak toward 1636 cm^−1^ and the amide II peak toward 1533 cm^−1^. This red shift is attributed to the formation of Schiff bases, which have a distinct peak at 1620 cm^−1^ and are an indicator of successful crosslinking of gelatin fibers [36,37,38]. Additionally, Raman spectroscopy verified these results by displaying an intensity increase in the C=N stretching vibration at 1664 cm^−1^, confirming an increase in Schiff bases (Figure 4B) [39].

Based on these results with respect to scaffold stability, morphology, and confirmation of successful crosslinking, we chose the conditions of 25% GA/90 min for further cell culture experiments.

### 3.2. Physical Characterization of Electrospun Scaffolds

**Figure 5:**
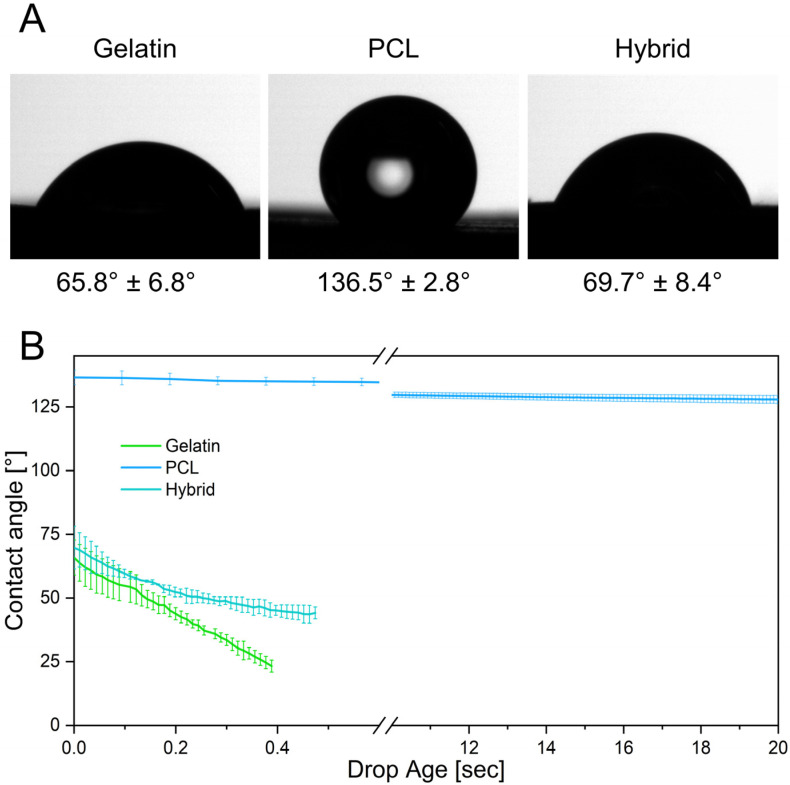
(**A**) Initial contact angles of gelatin, PCL, and the hybrid scaffold. (**B**) Contact angle measurements display the absorption of the water droplet into the hydrophilic scaffolds over time.

The biophysical characterization of the microenvironment provided by the fibrous cell culture scaffolds is of considerable interest because it directly influences the adhesion, proliferation, and differentiation of cells [40,41,42]. Contact angle measurements were conducted to evaluate the surface wettability of the prepared electrospun fiber scaffolds as a determinant for successful cell adhesion on surfaces. By measuring the contact angle of a single water droplet on the fiber mat, its hydrophilicity can be determined. To classify the surface wettability, contact angles of more than 90° are usually referred to as hydrophobic, whereas contact angles of less than 90° are considered hydrophilic [43]. Previous studies revealed that the optimal prerequisite for cell adhesion is provided by moderate hydrophilic conditions with initial contact angles ranging from 35° to 80° [44,45]. In contrast, more hydrophilic or hydrophobic surfaces led to a decrease in cell attachment and impeded subsequent cell migration [44,45]. The initial contact angles after the deposition of a water droplet onto the surfaces of different electrospun scaffolds are depicted in Figure 5A. Gelatin fibers display a contact angle of 65.8° ± 6.8°, exhibiting a higher degree of surface wettability due to their hydrophilic protein backbone and therefore providing more favorable properties as a cell culture substrate. In contrast, PCL exhibits a contact angle of 136.5° ± 2.8°, confirming the hydrophobicity of the polymer scaffold and indicating potentially impaired cell attachment. Interestingly, the hybrid scaffold with a ratio of 1:1 of gelatin and PCL fibers exhibits a contact angle of 69.7° ± 8.4°, attesting that the equal incorporation of both polymers is sufficient to tune the surface wettability to moderate hydrophilicity, which is favorable for cell adhesion.

Furthermore, the contact angle can be measured over time, elucidating dynamic interactions of the droplet with the surface of the fiber scaffolds. Here, rapid absorption of the deposited water droplet can be observed on the gelatin scaffold, as indicated by a decrease in contact angle over the measurement period. In contrast, the contact angles on the PCL scaffold only change slightly over 20 s, demonstrating the slow absorption and low wettability of PCL (Figure 5B). The hybrid scaffolds also exhibit a rapid decrease in contact angle as the water droplet seeps into the hydrophilic fiber network, emulating the dynamics observed in gelatin scaffolds. These kinetic contact angle measurements over an extended period of time reveal the water-resistant characteristics of PCL fibers by preventing water penetration into the scaffold, whereas the gelatin and hybrid scaffolds displayed rapid wetting throughout the fiber network. Due to the fast water uptake, these scaffolds are more likely to enable higher diffusion rates of solubles through the scaffold, improving the cellular crosstalk and nutrition of cells [46,47].

Besides excellent prerequisites for cell adhesion due to the hydrophilic surface, the electrospun scaffolds were further engineered to structurally resemble the native environment of the human BBB. The basement membrane, providing support for endothelial cells in the native BBB and a physical separation from the surrounding pericytes, constitutes an ultrathin structure, still allowing for crosstalk between the vascular and neuronal compartment. To replicate this membrane as a cell culture scaffold, it is necessary to fabricate electrospun scaffolds as thin as possible. The thickness of the different scaffolds was adjusted to 11.2 ± 3.4 µm, approximating the thickness of conventional Transwell^®^ inserts while still providing sufficient stability of the fibrous structure for cell culture applications. Handling of the ultrathin and flexible scaffolds during cell culture applications, e.g., insertion into CellCrown™ inserts, can be difficult. Therefore, we added a supporting layer of PVP fibers (approx. 175 µm thick) underneath the cell culture substrates, which are unaffected by crosslinking with GA and thus dissolve upon immersion in cell culture media. Hence, the PVP layer has a dual function by absorbing the water vapor in the crosslinking process and stabilizing the electrospun scaffolds to facilitate handling.

**Figure 6:**
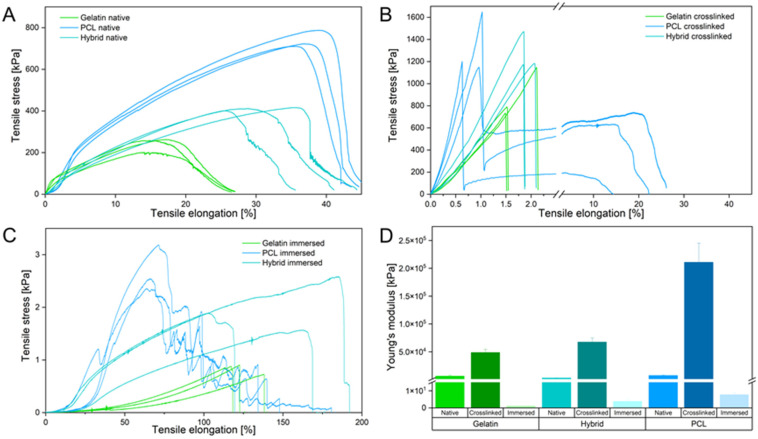
(**A**–**C**) Stress–strain curves of the native (**A**), crosslinked (**B**), and immersed (**C**) electrospun scaffolds. (**D**) Young’s modulus of native, crosslinked, and immersed electrospun scaffolds.

Because the mechanical microenvironment strongly influences the migration and proliferation of cells, comprehensive mechanical testing was carried out to determine the structural properties of the scaffolds [40]. Mechanical characterization of all scaffolds was performed via uniaxial tensile testing in three different stages of the fabrication process: before crosslinking (Figure 6A), after crosslinking with GA (25% *v*/*v*) for 90 min (Figure 6B), and after immersing the crosslinked fibers in PBS to assess the mechanical properties under cell-culture-like conditions (Figure 6C).

In their native state, gelatin fibers show weak mechanical properties by withstanding a maximum tensile stress of 258 kPa and elongation of about 20% until failing under a gradual fiber fracture. Each rupture event of the fibers led to a reorganization along the tensile axis, facilitating an increased elongation of the gelatin scaffold. In contrast to gelatin, PCL fibers resist four times higher tensile stress and are elongated twice as far as gelatin. The PCL fibers underwent a necking process along the tension axis until rapid crack propagation occurred. The hybrid scaffolds display an increase in resistible tensile stress and elongation compared to pure gelatin fiber mats. Interestingly, rupture characteristics similar to those of gelatin fibers were identified for the hybrid scaffold. Homogeneous distribution of PCL and gelatin fibers leads to a mechanically improved scaffold that combines the mechanical attributes of both fiber types. Compared to the native scaffolds, all crosslinked samples show an increase in brittleness, as indicated by the slope increase in the elastic region of the stress–strain curve and shorter elongation of fibers (Figure 6B). The elongation of all scaffolds decreased, whereas the maximum endured tensile stress increased due to the film formation of the supporting PVP layer by crosslinking the fibers in the vapor phase of an aqueous GA solution (see Supporting Information Figure S1). Notably, only the crosslinked PCL scaffold underwent a delamination process, in which the hydrophilic PVP film peeled off of the ductile and hydrophobic PCL fibers, resulting in a double-failure event displayed in the stress–strain curve (Figure 6B). The hydrophilic gelatin and hybrid scaffold expressed bulk-like failure properties by interacting with the PVP film. To assess the mechanical properties of the scaffolds in an in vitro relevant context, tensile strength was evaluated after immersing the fiber mats in PBS, which dissolved the PVP layer and hydrated the remaining fibers. The gelatin scaffold underwent extensive necking under low tensile stress and failed with a single failure event, emphasizing the structural weakness of the gelatin scaffold. In contrast to gelatin, defects of the PCL fibers appeared at multiple positions within the sample until a critical fiber defect density led to the failure of the scaffold. Like before, the hybrid scaffold combines the mechanical properties of both polymer fibers within one scaffold, displaying higher tensile strength and ductility compared to pure gelatin and PCL scaffolds, respectively. These mechanical characteristics displayed by the hybrid scaffold successfully imitate critical properties of collagen in the native ECM, where native collagen fibrils show a high ductility to prevent brittle-like failure events [48,49,50]. Furthermore, assessment of Young’s modulus (or elastic modulus), calculated as the slope in the elastic region of the stress–strain curves, provides additional information about the elasticity of materials and their resistance to deformation, enabling the comparison of the elasticity of the three scaffolds with the native ECM. Analysis of the elastic modulus revealed the importance of an adequate testing method for the mechanical characterization of electrospun scaffolds, displaying a variability between the immersed and crosslinked scaffolds in a magnitude of 1 × 10^5^. Compared to the native electrospun scaffolds, Young’s modulus confirmed the increase in brittleness after crosslinking due to the film formation of PVP by a 20-fold increase for each scaffold. The immersion of the electrospun scaffolds leads to a 1000-fold decrease in the elastic moduli compared to the native state, highlighting that analysis in different fabrication stages can lead to false assumptions regarding the mechanical properties of the scaffold. Only the immersed electrospun scaffolds, which show the mechanical behavior in vitro, are able to closely mimic the mechanical properties of the in vivo situation. For native human brain tissue, Young’s moduli of 0.5–4.5 kPa have been reported, whereas commercially available PET Transwell^®^ membranes display elastic moduli of about 2 GPa [51,52]. In contrast, the electrospun gelatin scaffold has a Young’s modulus of 1.09 ± 0.08 kPa, whereas PCL displays the highest elastic modulus of 7.68 ± 1.13 kPa. As before, the hybrid scaffold combines the structural properties of both polymers and exhibits an elastic modulus of 3.79 ± 1.14 kPa (Figure 6D). Considering the findings from the physical characterization of the electrospun scaffolds, we conclude that they have the potential to closely mimic the native BBB by resembling an adequate microenvironment with moderate hydrophilicity, low fiber mat thickness, and in vivo-like elasticity to foster cell adhesion, proliferation, and migration, respectively.

### 3.3. Cultivation of the Blood–Brain Barrier on Electrospun Scaffolds

**Figure 7:**
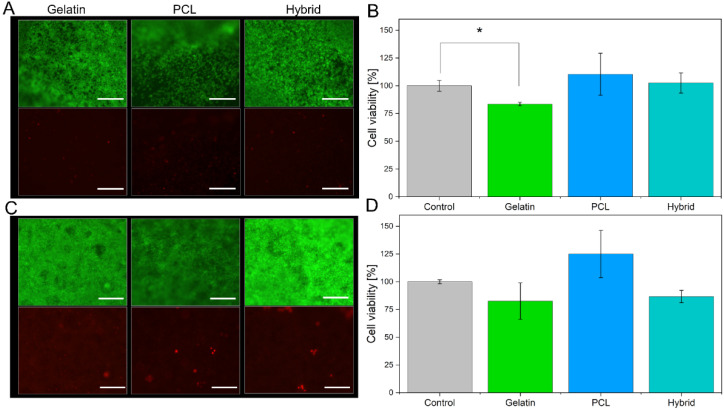
Live–dead staining of cells cultivated on all electrospun scaffolds, with living cells are shown in green (fluorescein diacetate, upper panel), and dead cells are shown in red (propidium iodide, lower panel), as well as the respective alamarBlue^®^ assays for hBMECs (**A**,**B**) and hiPS-BCECs (**C**,**D**). Transwell^®^ membrane inserts were used as a positive control. The inserts were collagen IV-fibronectin-coated for the cultivation of hiPS-BCECs. Scale bars represent 500 µm (**A**) and 200 µm (**C**). * *p* < 0.05 for gelatin scaffolds compared to the control.

To evaluate the suitability of gelatin, PCL, and hybrid fibers as cell culture scaffolds, we cultivated the immortalized human brain capillary endothelial cell line (hBMEC) on the different fiber formulations for 48 h and assessed the cell viability. Neutralization of the remaining GA from the crosslinking procedure was carried out with a 2 M glycine solution to minimize cytotoxic effects on the cells. Among the three electrospun scaffolds, different cell densities were observed during live–dead staining (Figure 7A). PCL scaffolds, which have hydrophobic surface characteristics and therefore hamper cell adhesion of hBMECs, display a lower cell density. In contrast, the electrospun scaffolds containing gelatin (gelatin and hybrid) exhibit higher cell densities on the surface of the electrospun scaffold due to favorable physical properties, such as moderate hydrophilicity and optimized biomechanics of the cell substrates. Furthermore, live–dead staining revealed the high suitability of all electrospun scaffolds based on the small number of dead cells indicated by the red fluorescence of propidium iodide (Figure 7A, lower panel). To confirm these results, we assessed the metabolic activity of the cell monolayer cultured on the three scaffolds by conducting an alamarBlue^®^ assay and comparing the findings to cells cultured in Transwells^®^ as a control group (Figure 7B).

Here, the cell viability for cells cultured on either of the three tested electrospun scaffolds is more than 80%, confirming the suitability of all cell substrates. However, lower fluorescence intensity in the alamarBlue^®^ assay was observed for cells cultivated on gelatin scaffolds compared to PCL scaffolds, indicating that the metabolic activity of the hBMECs is reduced. This effect is induced by the higher cell density on the gelatin scaffold, as shown by the live–dead staining; a higher cell density results in increased cell–cell contacts, which in turn reduces the metabolic activity of the cell monolayer [53]. The reduction in metabolic activity decreases the amount of converted resazurin to resofurin, leading to seemingly lower cell viability.

After confirming the suitability of the electrospun scaffolds for the cultivation of hBMECs, hiPS-BCECs were cultivated on the nanofibrous systems. Usually, the cultivation of these cells on Transwell^®^ membrane inserts requires a coating consisting of collagen IV and fibronectin, components of the human basement membrane, in order to enable the adhesion and subsequent proliferation of hiPS-BCECs [16,54]. However, hiPS-BCECs were found to adhere to all electrospun scaffolds without an additional coating (Figure 7C), confirming that the microstructure of the electrospun scaffolds (such as fiber diameter, moderate hydrophilicity, and biomechanics) successfully mimics the native physicochemical properties of the ECM. Cell viability assessment of hiPS-BCECs cultured on electrospun scaffolds was carried out using live–dead staining and an alamarBlue^®^ assay. As a positive control, cells were cultured on Transwell^®^ membrane inserts coated with a collagen IV and fibronectin solution, representing the current standard for cultivating in vitro hiPSC-based BBB models. The results of the live–dead staining of the hiPS-BCECs are comparable to the results obtained from hBMECs cultures. All electrospun scaffolds display high cell viability based on the confluent monolayer of living cells (green staining) and small numbers of dead (red-stained) cells (Figure 7C). Notably, the same tendencies with respect to metabolic activity and associated cell viability as observed in hBMEC cultures were observed, as shown in Figure 7D. Cultures of hiPS-BCECs on PCL scaffolds showed increased metabolic activity compared to the positive control by 125%, whereas the cultivation of cells on gelatin and hybrid scaffolds resulted in viabilities of about 80% and 86.7%, respectively. Although cell viability of the electrospun scaffolds was lowered compared to the Transwell^®^ control group, a viability of more than 80% classifies the scaffolds as adequate substrates for culturing hBMECs and hiPS-BCECs. With respect to the coating-independent attachment of hiPS-BCECs to the nanofibers, the electrospun scaffolds represent a promising alternative to collagen IV-fibronectin-coated Transwell^®^ inserts.

### 3.4. Immunofluorescence Imaging of Tight Junctions

**Figure 8:**
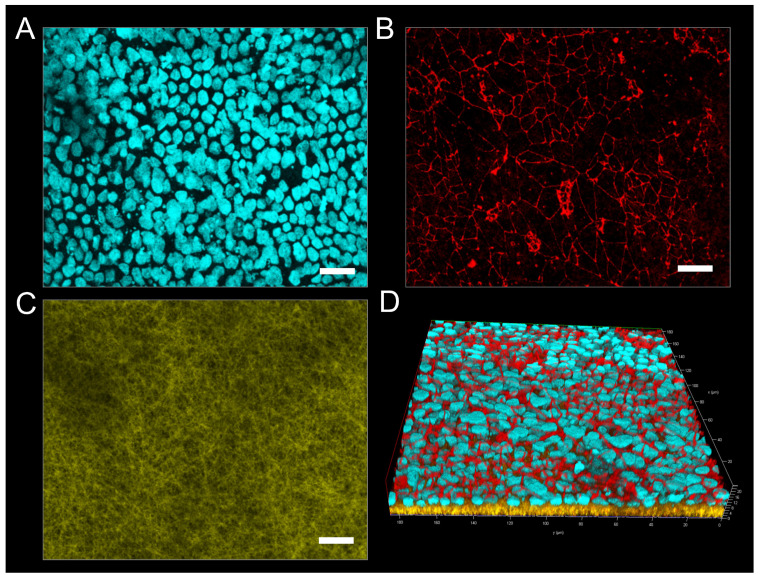
(**A**–**C**) Representative confocal immunofluorescence images of hiPS-BCECs on gelatin fibers. hiPS-BCECs were stained for cell nuclei (cyan) (**A**), displaying occluding (red) (**B**) on electrospun fibers. (**D**) Z-stack images of hiPS-BCECs on electrospun gelatin scaffolds. Scale bars represent 20 µm.

One of the essential proteins associated with the formation and functionality of the BBB is the tight junction protein occludin, which is expressed on the cell surface of endothelial cells connecting neighboring cells to each other, thus separating microvasculature blood from the extracellular fluid of the central nervous system [55]. Liebner et al. showed that the expression, morphology, and correct localization of tight junctions influence the barrier characteristics, such as a high transendothelial electrical resistance (TEER) and low permeability [56]. Therefore, the visualization of occludin represents a common determinant of adequate barrier formation. In this respect, the endothelial monolayers were stained for cell nuclei (cyan) (Figure 8A) and occludin (red) (Figure 8B) and subsequently visualized by fluorescence microscopy (Figure 8). Additionally, elucidating the complex interplay of the endothelium with the electrospun scaffolds, fluorescent PCL fibers were created by incorporating sodium fluorescein, whereas gelatin fibers were visualized via their distinct autofluorescence after crosslinking with GA (both yellow, see Supporting Information Figure S2). Therefore, both fiber types could be simultaneously excited at a wavelength of 488 nm, allowing for visualization of the complete fibrous scaffold during immunostaining (Figure 8C). The acquired confocal images reveal the formation of a continuous cell monolayer of the hiPS-BCECs on the surface of each scaffold.

Here, tight junctions are expressed on the entire monolayer at the interface between neighboring cells. To further evaluate the organization of tight junctions in the three-dimensional context of the endothelial barrier, z-stack fluorescence images were acquired, and three-dimensional renderings of the tissue cultures were created (Figure 8D). The hiPS-BCECs on the gelatin scaffold are localized on the surface of the nanofibers, forming a dense barrier by apically expressing occludin, which correlates with the physiological localization of tight junction proteins in the human BBB (Figure 8D) [56].

In contrast to the gelatin scaffold, hiPS-BCECs did not sufficiently adhere on the PCL scaffold; therefore, acquisition of immunofluorescence images was not possible. On the hybrid scaffold, hiPS-BCECs are localized on the surface of the fiber mat, displaying improved apical occludin expression at the cell–cell interfaces, complying with the in vivo situation. The improved occludin expression in scaffolds containing gelatin can be explained by the decreased fiber diameters, which allow for reduced pore sizes within the scaffolds, favoring faster cell spreading instead of cell migration into the scaffold, accelerating barrier formation [25,57]. Furthermore, the use of gelatin fibers is necessary for improved mimicry of the basement membrane, resulting in a stronger adhesion of the cellular monolayer onto the electrospun scaffolds. The occludin localization is most concise for hiPS-BCECs cultured on gelatin fibers, representing the closest mimicry of the in vivo localization of tight junctions in the BBB, followed closely by the hybrid scaffold. The use of gelatin nanofibers also improved the polarization of cells and thus the localization of tight junctions. The correct expression of occludin directly correlates with the barrier functionality of the cell monolayer because parameters such as the electrical resistance or paracellular permeation of compounds are mainly dependent on the number and complexity of tight junctions in the endothelium [58].

### 3.5. Evaluation of Barrier Properties

Besides visualization of tight junctions, barrier integrity was further analyzed via the measurement of TEER values and the permeability of sodium fluorescein. Analytical assessment of TEER is a well-established method to quantify the integrity of the barrier in in vitro or ex vivo models [59]. The TEER value of the human native BBB in vivo is estimated to be more than 2000 Ω × cm^2^ [8,12]. When cultivated on Transwell systems, in vitro BBB models reach high TEER values, as observed in this study, in which Transwell^®^ models were used as a control, reaching TEER values of 3218.32 ± 340.57 Ω × cm^2^. However, studies have shown that the relationship between TEER values and the permeability of BBB models is not linear, and consensus values are reached in the scientific community, depending on the cellular origin of the BBB model cells [60]. For hiPSC-derived BBB models, a minimum threshold of 500 Ω × cm^2^ has repeatedly been achieved and reported to sufficiently mimic the native BBB [52].

**Figure 9:**
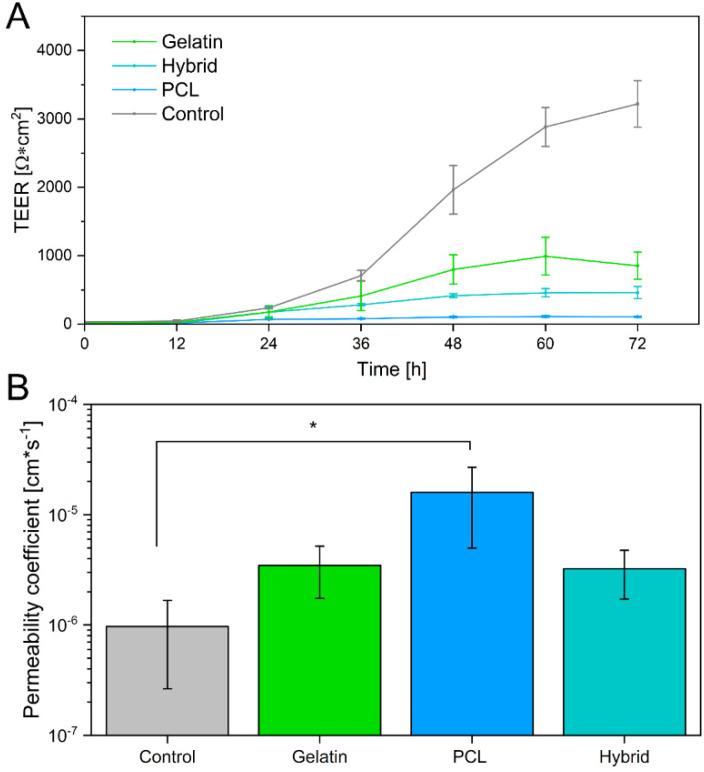
(**A**) Transendothelial electrical resistance of hiPS-BCECs cultivated on gelatin, hybrid, and PCL electrospun scaffolds, as well as Matrigel^®^-coated Transwell^®^ inserts. (**B**) Permeability of sodium fluorescein across cultivated hiPS-BCECs on electrospun scaffolds. Matrigel^®^-coated Transwell^®^ inserts were used as a control. * *p* < 0.05 for PCL compared to the control.

Few individual studies on in vitro BBB models incorporating electrospun fibers as already exist. However, these models exhibited TEER values in the range of 10–250 Ω × cm^2^, displaying weak barrier properties [51,61,62,63]. In this study, TEER values were measured over a cultivation time of 72 h. Generally, the TEER values of hiPS-BCECs on all electrospun scaffolds increased over 72 h. Nevertheless, the characteristics of each individual scaffold strongly influenced the final barrier properties of the cell layer, as shown in Figure 9A. As expected based on the immunostaining results, hiPS-BCEC cultivated on PCL scaffolds presented the lowest TEER values, making them the weakest barrier. The maximum TEER values (113 ± 12 Ω × cm^2^) were reached after 60 h and remained constant afterward, consequently excluding the PCL-based scaffold as an adequate cell culture substrate. In contrast, the TEER values of hiPS-BCECs on gelatin and hybrid scaffolds reached a maximum of 946 ± 404 Ω × cm^2^ and 461 ± 87 Ω × cm^2^ after 72 h, respectively, exceeding the values published in the literature to date.

Because it has been shown that the TEER value itself is not sufficient to reliably assess the barrier integrity of the formed BBB, we further investigated the paracellular drug transport by quantifying the permeability of sodium fluorescein, which is passively transported through the BBB [64,65]. Based on previous studies, the permeability coefficient for adequately emulating the human BBB is expected to be in the magnitude of 10^−6^ cm × s^−1^, which is achieved by the Transwell^®^-based control [66,67]. In contrast, the permeability coefficient of the formed endothelial barrier on the PCL scaffold exceeds this limit, displaying a permeability of 1.59 × 10^−5^ cm × s^−1^, which is 16 times higher than the required permeability (Figure 9B), further corroborating the unsuitability of this scaffold for models mimicking the barrier properties of the BBB. The permeability of sodium fluorescein decreases for scaffolds containing gelatin as a fiber substrate to 3.46 × 10^−6^ cm × s^−1^ and 3.23 × 10^−6^ cm × s^−1^ for endothelial barriers cultivated on gelatin and hybrid scaffolds, respectively. As previously mentioned, these results highlight the non-linear relationship between TEER values and the permeability of the BBB. Interestingly, although the models cultivated on the hybrid scaffolds exhibit TEER values of only 50% compared to the gelatin fiber models, both in vitro models show the same permeability of sodium fluorescein. In the same way, when comparing the permeability of these scaffolds to the Transwell^®^-based models, it becomes apparent that the permeability of the positive control does not significantly differ from that of in vitro models cultivated on electrospun gelatin-containing cell culture substrates, despite the significantly higher measured TEER values of the Transwell^®^ models. By incorporating gelatin fibers in the electrospun scaffolds, the barrier tightness increases and reaches values comparable to those of the Transwell^®^-based model and the native BBB, rendering hybrid scaffolds the most promising candidates for culturing in vitro BBB models to adequately mimic the in vivo properties of the human BBB.

## 4. Conclusions

In summary, we designed and characterized an in vitro model of the human blood–brain barrier by cultivating hiPS-BCECs on three-dimensional electrospun fiber scaffolds. By carefully selecting the polymers considering the structural stability, cell compatibility, and finetuning the electrospinning process, we achieved a high level of similarity with respect to fiber diameter, pore size, and elasticity in comparison to the native ECM. We preserved the fiber morphology of gelatin nanofibers during the crosslinking process and further enabled the creation of ultrathin, manageable electrospun scaffolds by incorporating a water-soluble protecting PVP base layer. The resulting biophysical properties of these systems closely resemble the natural basement membrane, providing a suitable cell culture substrate for the adhesion and proliferation of hiPSCs without the necessity of artificial membrane coatings. The electrospun scaffolds enabled the successful formation of a cellular monolayer, revealing an in vivo-like expression of tight junctions and resulting in the tightest blood–brain barrier formation on electrospun scaffolds published to date. Furthermore, the non-linear relationship between TEER value and barrier properties of the BBB was revealed, emphasizing that the presented hybrid scaffold is an adequate simulation of the basement membrane. Our novel nanofibrous system also provides a promising platform for the cocultivation of endothelial cells with pericytes or astrocytes, as the fiber structure allows for enhanced cellular crosstalk, further increasing its in vivo relevance.

## Figures and Tables

**Table 1:** Effect of various crosslinking conditions on gelatin nanofiber stability under aqueous conditions. The symbols indicate instable fibers (X), semistable fibers ((✓)), and fibers that were stable for more than one week (✓).

GA Concentration (*v*/*v*)/ Crosslinking Time	5%	15%	25%
60 min	X	(✓)	(✓)
90 min	X	(✓)	✓
120 min	X	(✓)	✓

## Data Availability

The data presented herein are available upon request from the corresponding author.

## Supplementary Materials

The following supporting information can be downloaded at: https://www.mdpi.com/article/10.3390/pharmaceutics14061308/s1, Figure S1: Morphological changes of PVP after crosslinking; Figure S2: Release of sodium fluorescein from PCL fibers.
