# Supplementary material for: Electrospun Scaffolds as Cell Culture Substrates for the Cultivation of an In Vitro Blood–Brain Barrier Model Using Human Induced Pluripotent Stem Cells
Source: Pharmaceutics. 2022 Jun 20;14(6):1308. doi: 10.3390/pharmaceutics14061308 (PMC9231001; doi:10.3390/pharmaceutics14061308)
Supplement: Supplementary file 1 [file pharmaceutics-14-01308-s001.zip › pharmaceutics-1774582-supplementary.pdf]

1 Supporting Information

1.1 PVP fibers before and after crosslinking

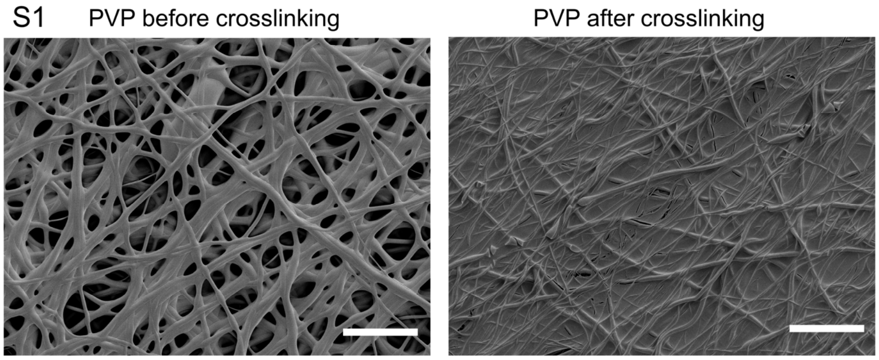

Figure S1: PVP before and after crosslinking with an aqueous GA solution. Scale bars represent 20 µm.

1.2 Release of sodium fluorescein from PCL fibers

S2

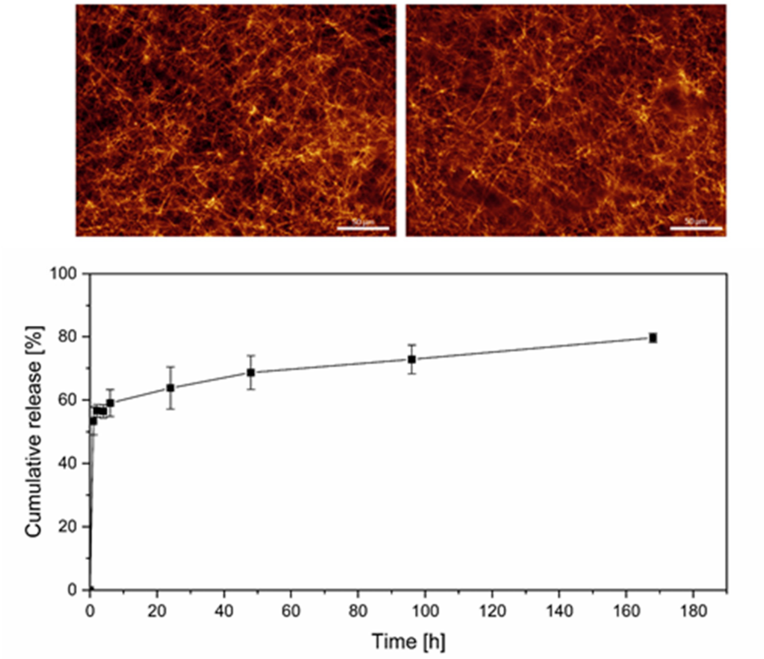

Figure S2: Images of fluorescein loaded PCL fibers after 2 (left) and 4 (right) weeks of incubation. Quantification of released sodium fluorescein from PCL fibers.

Fluorescein was incorporated in PCL fibers to visualize the fibers with fluorescence microscopy, elucidating the complex interplay of fibers and adhering cells. The substance was chosen due to its characteristics such as low toxicity, low costs, high fluorescence intensity, and a fluorescence spectra similar to the spectra of crosslinked gelatin. After immersion of the PCL fibers in PBS for 2 and 4 weeks, fluorescence microscopy images were acquired, and PCL fibers were successfully visualized. The fibers do not exhibit a decrease in fluorescence intensity after 4 weeks, thus enabling the use as fluorescently labeled *in vitro* cell culture substrate. Nevertheless, by incubating the scaffolds in cell culture media, a release of fluorescein occurs, thus making it necessary to determine the release of fluorescein, excluding any cytotoxic fluorescein concentrations within the cell culture medium. The release of

fluorescein is characterized by a burst release after 2 hours due to adhered dye on the fiber surface. However, this burst release takes place during the incubation with glycine solution, which is exchanged after 4 hours, reducing the amount of fluorescein significantly. Afterward, a steady release is achieved over 7 days with a maximum released amount of 79.64% $\pm$ 1.43%. We conclude that the released fluorescein does not influence the adhesion and proliferation of hiPSCs and is, therefore, suitable for visualizing PCL fibers.
